# Comparative analysis of the rhizosphere microbiome and medicinally active ingredients of *Atractylodes lancea* from different geographical origins

**DOI:** 10.1515/biol-2022-0769

**Published:** 2023-11-23

**Authors:** Junjie Tang, Yun Han, Lingfeng Pei, Wei Gu, Rongli Qiu, Sheng Wang, Qihan Ma, Yifu Gan, Min Tang

**Affiliations:** School of Pharmacy, Nanjing University of Chinese Medicine, 138 Xianlin Avenue, Qixia District, Jiangsu, Nanjing, 210023, China; Suzhou TCM Hospital Affiliated to Nanjing University of Chinese Medicine, Suzhou, 215002, China; Jiangsu Collaborative Innovation Center of Chinese Medicinal Resources Industrialization, Nanjing, 210023, China; State Key Laboratory of Dao-di Herbs, Beijng, 100700, China

**Keywords:** *Atractylodes lancea*, microbiology, medicinally active ingredients, metagenomics

## Abstract

This study aimed to explore the important role of the rhizosphere microbiome in the quality of *Atractylodes lancea* (Thunb.) DC. (*A. lancea*). The rhizosphere microbial community of *A. lancea* at two sampling sites was studied using metagenomic technology. The results of α-diversity analysis showed that the rhizosphere microbial richness and diversity were higher in the Maoshan area. The higher abundance of core microorganisms of the rhizosphere, especially *Penicillium* and *Streptomyces*, in the Maoshan area compared with those in the Yingshan area might be an important factor affecting the yield of *A. lancea*. Redundancy analysis illustrated that the available phosphorus had a significant effect on the rhizosphere microbial community structure of *A. lancea*. We also showed that the plant–microbe and microbe–microbe interactions were closer in the Maoshan area than in the Yingshan area, and *Streptomyces* were the main contributors to the potential functional difference between the two regions. *A. lancea* in the Maoshan area had a high content of atractylodin and atractylon, which might be related to the enhanced abundance of *Streptomyces*, *Candidatus-Solibacter*, and *Frankia*. Taken together, this study provided theoretical insights into the interaction between medicinal plants and the rhizosphere microbiome and provides a valuable reference for studying beneficial microbes of *A. lancea*.

## 1 Introduction

The *Atractylodes lancea* (Thunb.) DC. rhizome, also known as “Maocangzhu” or “Nancangzhu,” is a well-known traditional Chinese medicine (TCM) with high medicinal value, which is traditionally used in clinics to treat night blindness, digestive disorders, and influenza [1,2,3]. Maoshan in Jiangsu Province is traditionally considered a genuine producing area; *A. lancea* from Maoshan is regarded as having the best quality, as evidenced by long-term clinical practice. *A. lancea* is rich in volatile oil, which is mainly composed of sesquiterpenoids and polyacetylene compounds [4,5]. However, the contents of atractylodin, atractylon, and β-eudesmol of *A. lancea* from different producing areas show large variations [6,7]. The rhizosphere microbiome, geographical location, and climate are important factors that affect the formation of TCM. The rhizosphere microbes exert an important influence on plant growth and secondary metabolite formation [8,9,10]. Therefore, one factor contributing to the differing contents of medicinally active ingredients could be variation in the rhizosphere microbiome from different geographical origins.

The composition of microbial communities in the rhizosphere is markedly different from that in bulk soil. This phenomenon is referred to as the “rhizosphere effect” [11]. Studies have revealed the significant influence of the rhizosphere microbiome on plant growth, which includes nitrogen-fixing bacteria, plant growth-promoting rhizobacteria, biological control bacteria, and disease-suppressing bacteria [12]. In addition, researchers have discovered that particular plant–microbial interactions promote the production of active ingredients in medicinal plants, possibly because certain microbes contain a gene pool encoding proteins that are involved in the biosynthesis of active ingredients, thereby providing the host with additional metabolic capacity [13,14]. For example, the unique microbiome in the seeds of *Salvia miltiorrhiza* Bge is rich in functions related to secondary metabolism, which might influence tanshinone biosynthesis [15]. Co-culture of immobilized spores of *Aspergillus niger* and *Panax ginseng* adventitious roots significantly increased the ginsenoside content in the roots of *Panax ginseng* [16].

Since the first metagenomic data were published, researchers have begun to analyze the rhizosphere microbial diversity, community structure, interactions, and gene functions based on metagenomic techniques [17,18,19,20]. However, there have been few studies on the rhizosphere microbiome of *A. lancea*, and a comparative evaluation of the rhizobiome composition in different regions is still lacking [21]. Metagenomics now provides an effective platform to explore the structure and function of the rhizosphere microbiome of *A. lancea* in different regions. Therefore, in the present study, a metagenomic approach was used to analyze the differences in the microbiome community structure, function, and medicinally active ingredients of *A. lancea*. This study aimed to link the better medicinal quality of *A. lancea* with its specific rhizosphere microbiome to provide a new understanding of how the rhizosphere microbiome can enhance the production and quality of *A. lancea*.

## 2 Materials and methods

### 2.1 Sample collection

The rhizosphere soil was collected from Maoshan (Mao), Jiangsu Province (119°16′50″E, 31°42′37″N, and altitude 72.10 m) and Yingshan (Ying), Hubei Province (115°46′56″E, 31°3′28″N, and altitude 416.41 m) in July 2018. Each sample was collected using the five-point sampling method, and based on the definition of rhizosphere soil (soil close to the root surface) [22], a sterile plastic shovel was used to collect rhizosphere soil samples. The five individual soil samples collected were mixed and homogenized into one pooled sample, and in each plot, samples were repeated three times, which were denoted M1, M2, and M3 from Maoshan; and Y1, Y2, and Y3 from Yingshan, respectively. Similarly, corresponding bulk soil samples were collected. All soil samples were stored on dry ice immediately after collection and stored at −80°C until further analysis. The rhizosphere soil was used for DNA extractions and bulk soil for measurements of physicochemical parameters. Fresh *A. lancea* were dried at 45°C in an oven (DHG-9140A, Jinghong, Shanghai, China) to constant weight, and then crushed and filtered through a ≤2 mm sieve for further detection.

### 2.2 Physicochemical parameters of the soil

The physicochemical properties of the soil were determined as follows. The pH was determined using a PHS-3E pH meter (INESA Scientific Instrument Co., Ltd, Shanghai, China). About 10.00 g of air-dried soil was sieved through a 1 mm sieve and placed in a conical bottle. Then, 25 mL of water without CO_2_ (boiling for 10 min and then cooling) was added and sealed with a sealing film. It was placed in an oscillator for 2 min; after standing for 30 min, the pH of the soil suspension was measured. The available nitrogen (AN), available phosphorus (AP), and available potassium (AK) were quantified using a TPY-8A Soil Nutrient tester (Topu, Zhejiang, China) according to the manufacturer’s instructions.

### 2.3 DNA extraction and sequencing

Total genomic DNA was extracted from the six pooled samples using the MP Soil DNA extraction kit (Yanhuibio, Shanghai, China) according to the manufacturer’s instructions. The concentration and purity of the extracted DNA were determined with TBS-380 and NanoDrop2000 (Thermo, MA, USA) at 260/230 and 260/280 nm, respectively. The quality of the DNA extract was checked by 1% agarose electrophoresis. The paired-end library was constructed using NEXTFLEX Rapid DNA-Seq (Bioo Scientific, Austin, TX, USA). Adapters containing the full complement of sequencing primer hybridization sites were ligated to the blunt end of the fragments. Paired-end sequencing was performed on Illumina Novaseq 6000 (Illumina Inc., San Diego, CA, USA) at Majorbio Bio-Pharm Technology Co., Ltd. (Shanghai, China) using NovaSeq Reagent Kits according to the manufacturer’s instructions. Data processing was performed using the online Majorbio Cloud Platform (www.majorbio.com).

### 2.4 Bioinformatics analysis

Fastp software (version 0.20.0) was used to remove low-quality reads and those reads containing N among the raw sequences, while high-quality pair-end reads and single-end reads were retained. All cleaned sequences were assembled using MEGAHIT (version 1.1.2). Contigs with a length ≥300 bp were selected as the final assembling result, and they were used for further gene prediction and annotation. Open reading frames (ORFs) from each assembled contig were predicted using MetaGene. Then, a non-redundant gene catalog was constructed using CD-HIT (version 4.6.1). High-quality reads were aligned to the non-redundant gene catalogs to calculate gene abundance with 95% identity using SOAPaligner (version 2.21).

Representative sequences of the non-redundant gene catalog were aligned to the NR database using Diamond (version 0.8.35) for taxonomic annotations (*e*-value ≤ 1 × 10^−5^). Annotation of a cluster of orthologous groups (COGs) of proteins for the representative sequences was performed using Diamond against the eggNOG database (*e*-value ≤ 1 × 10^−5^). The KEGG annotation was conducted using Diamond against the Kyoto Encyclopedia of Genes and Genomes database (*e*-value ≤ 1 × 10^−5^).

### 2.5 Sample preparation and analytical procedure

About 1.0 g of the sample powder was weighed; then, it was sonicated with methanol (25.0 mL) for 60 min, cooled, made up for the weight loss with methanol, shaken well, and then filtered through a 0.45 μm filter. The three medicinally active ingredients of samples were determined using high-performance liquid chromatography (HPLC) (Waters e2695, USA). The separation was conducted using an Inertsil™ ODS-3 column (4.6 mm × 250 mm, 5 μm) at 30°C and the injection volume was 10 μL. Mobile phase A contained 0.1% formic acid in acetonitrile, and mobile phase B contained 0.1% formic acid in water at 0.8 mL/min flow rate with the following gradient elutions: 0–5 min, 52–60% A; 5–40 min, 60–75% A; 40–44 min, 75–80% A; 44–57 min, 80–52% A. Atractylodin, atractylon, and β-eudesmol were detected at 340, 220, and 203 nm, respectively.

For the generation of standard curves, the standards of atractylodin (Lot: B20128, HPLC ≥ 98%, Shanghai yuanye Bio-Technology Co., Ltd), atractylon (Lot: B24634, HPLC ≥ 95%, Shanghai yuanye Bio-Technology Co., Ltd), and β-eudesmol (Lot: B21969, HPLC ≥ 95%, Shanghai yuanye Bio-Technology Co., Ltd) were prepared at concentrations of 0.601, 0.620, and 0.920 mg/mL. After gradient dilution, 10 μL of the three standards were injected and detected at 340, 220, and 203 nm, respectively. The peak area values of each standard were recorded (Figure S1). The linear regression equations were then calculated using the sample concentration of standards as abscissa (*X*) and the peak areas as ordinate (*Y*) (Figure S2). The peak value of each active ingredient was substituted into the linear regression equation to obtain the concentration of the sample and then multiplied by dilution to calculate the amount of active ingredients in the sample (mg/g).

### 2.6 Data analysis

The Chao, Shannon, and Simpson indexes were used to evaluate the *α-*diversity of the sample [23,24,25], for which differences were tested by independent sample *T-*test (*P* ≤ 0.05 indicates statistical significance). The β-diversity of rhizosphere microorganisms among samples was analyzed by principal coordinate analysis (PCoA). A two-sided Welch’s *T*-test was applied to test the differences in microbial communities and functions among Mao and Ying samples (*P* ≤ 0.05 indicates statistical significance). Redundancy analysis (RDA) was used to evaluate the correlation between rhizosphere microorganisms and environmental factors. The data on the annual average temperature (AAT) and annual average precipitation (AAP) were obtained from www.xihe-energy.com. The heat maps of KEGG functional pathways were processed by *z*-score normalization based on relative abundances. The data of this study were analyzed using IBM SPSS Statistics (version 26.0). For the analysis of the three active ingredient contents of different samples, the independent sample *T*-test was conducted, and OriginPro 2023 (version 10.0.0.154) was used to plot the graphs.

## 3 Results

### 3.1 Sequencing results

The number of raw reads obtained from the six rhizosphere soil-pooled samples of *A. lancea* was between 51,675,168 and 58,520,292. After quality control, the amount of raw sequencing data per group was reduced to an average of 99.45%, which indicates that the sequencing quality is high. The assembly of reads using MEGAHIT software generated an average of 562,714 and 293,043 contigs from the Mao and Ying samples, respectively. Their N50 lengths ranged from 461 to 634 bp, with an N90 between 325 and 348 bp. Two values of N50 and N90 were used to evaluate the effect of splicing assembly, and the larger the two values, the better the effect of splicing assembly. The ORFs in these contigs were predicted and used to construct non-redundant gene sets. Thus, a total of 4,406,678 catalog genes were generated from the metagenomic libraries.

### 3.2 Diversity analysis of the rhizosphere microbiome

**Figure 1:**
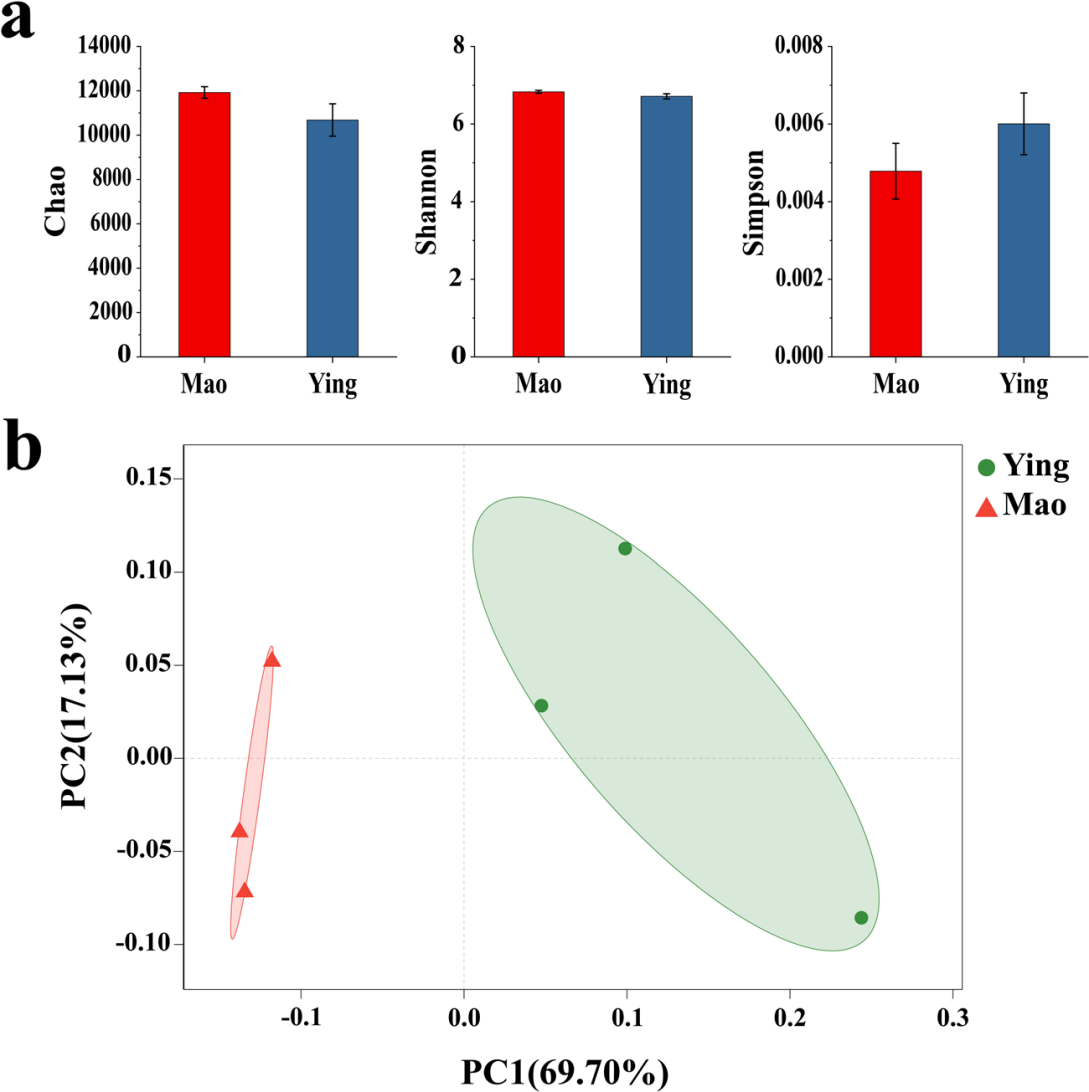
Diversity comparisons of the *A. lancea* rhizosphere microbiome. (a) α-Diversity in the rhizosphere microbiome was measured using the Chao, Shannon, and Simpson index. Chao: uses the chao1 algorithm to estimate the index of the number of OTUs contained in the sample. Shannon: one of the indices used to estimate microbial diversity in a sample; the higher the Shannon value, the higher the community diversity. Simpson: one of the indices used to estimate microbial diversity in a sample; the higher the Simpson index value, the lower the community diversity. (b) PCoA was based on the Bray–Curtis dissimilarity matrix to demonstrate the β-diversity of the rhizosphere microbiome.

The Chao, Shannon, and Simpson indexes represent microbial community richness and diversity, respectively. According to the Chao analysis, the Mao and Ying samples had 11,922 and 10,682 OTUs (operational taxonomic units, which is a basic unit for species classification and relative abundance analysis), respectively. The OTU richness of the Mao sample was higher than that of the Ying sample but the difference was not significant (*P* ≥ 0.05) (Figure 1a). The Shannon index correlates positively with microbial community diversity, while the Simpson index correlates negatively with microbial community diversity. According to these two indices, the microbial community diversity in the Mao sample was higher than that in the Ying sample but not significant. PCoA based on the Bray–Curtis distance also revealed that the rhizosphere microbial communities in the Mao and Ying samples were obviously separated (Figure 1b). The two main coordinates explained 86.83% of the microbial community changes among all the samples, of which the PC1 and PC2 axes explained 69.70 and 17.13% of the results, respectively.

### 3.3 Comparative analysis of microbial communities

**Figure 2:**
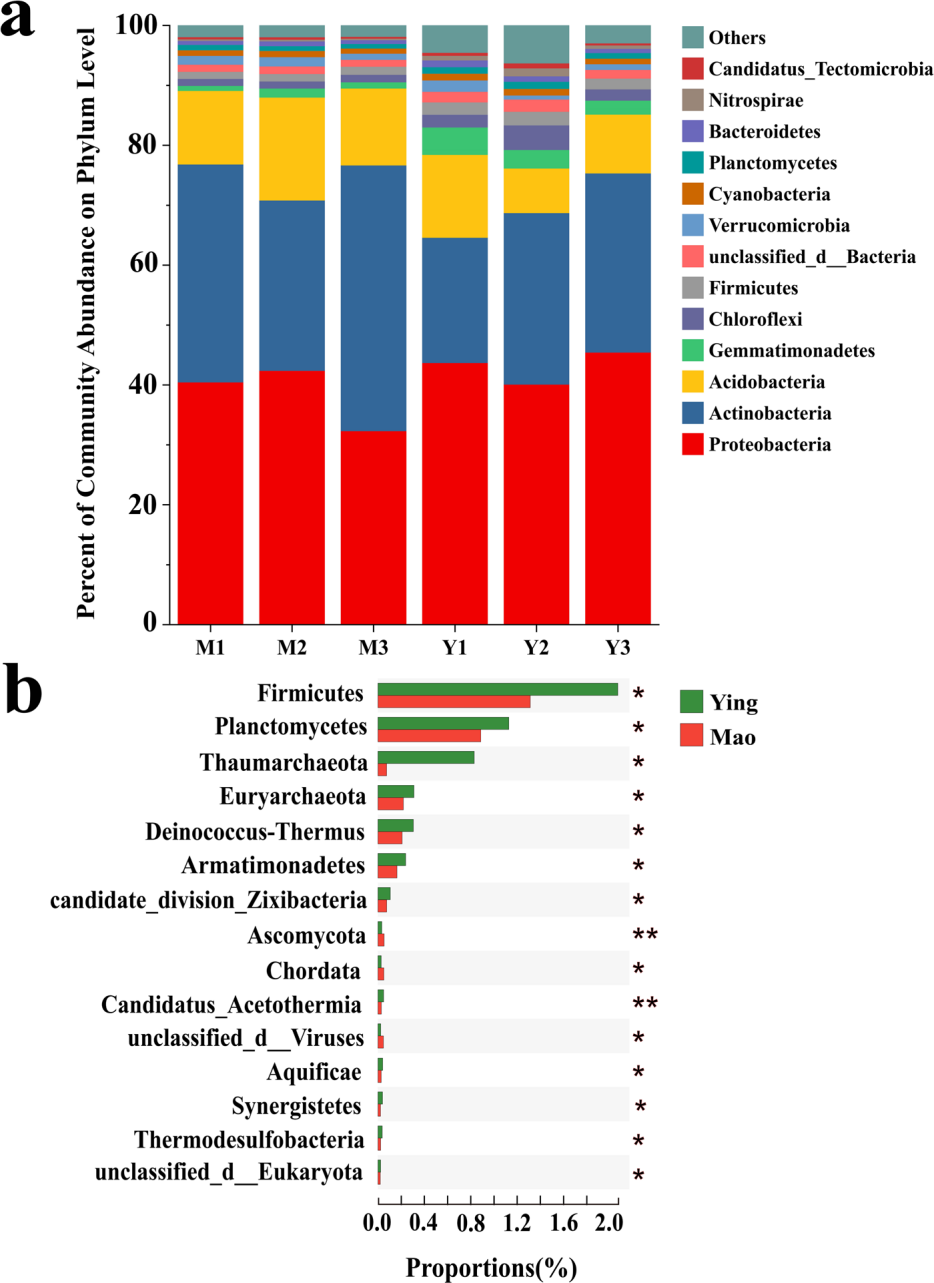
A comparison of procaryotic communities in the rhizosphere. (a) Relative abundance of the most abundant prokaryote at the phylum level. (b) Significant discrepancies in indicator prokaryotes between Mao and Ying groups at the phylum level. *P ≤ 0.05, **P ≤ 0.01.

Comparing the catalog gene sets with the NR database, we found that the abundance of bacteria in all samples was the highest, above 98%, followed by archaea. Prokaryotes accounted for more than 99% of the species in each sample, and each sample was annotated to an average of 0.25% eukaryotes. Based on the dominant role of prokaryotes in the rhizosphere microorganisms of *A. lancea*, this study focused on prokaryotes. A total of 123 phyla were identified in the six samples. The dominant phyla were Proteobacteria (39.35%), Actinobacteria (33.85%), Acidobacteria (13.12%), Gemmatimonadetes (1.85%), Chloroflexi (1.58%), and Firmicutes (1.50%) (Figure 2a), which comprised 90% of all OTUs and were well represented. Comparing the relative abundance of prokaryotes among the two sampling sites at the phylum, family, and genus level revealed a marked difference in the microbial communities between the Mao and Ying samples. At the phylum level, there were significant differences in the abundance of Firmicutes, Planctomycetes, Thaumarchaeota, Euryarchaeota, Deinococcus-Thermu, and Armatimonadetes between the Mao and Ying samples (Figure 2b).

**Figure 3:**
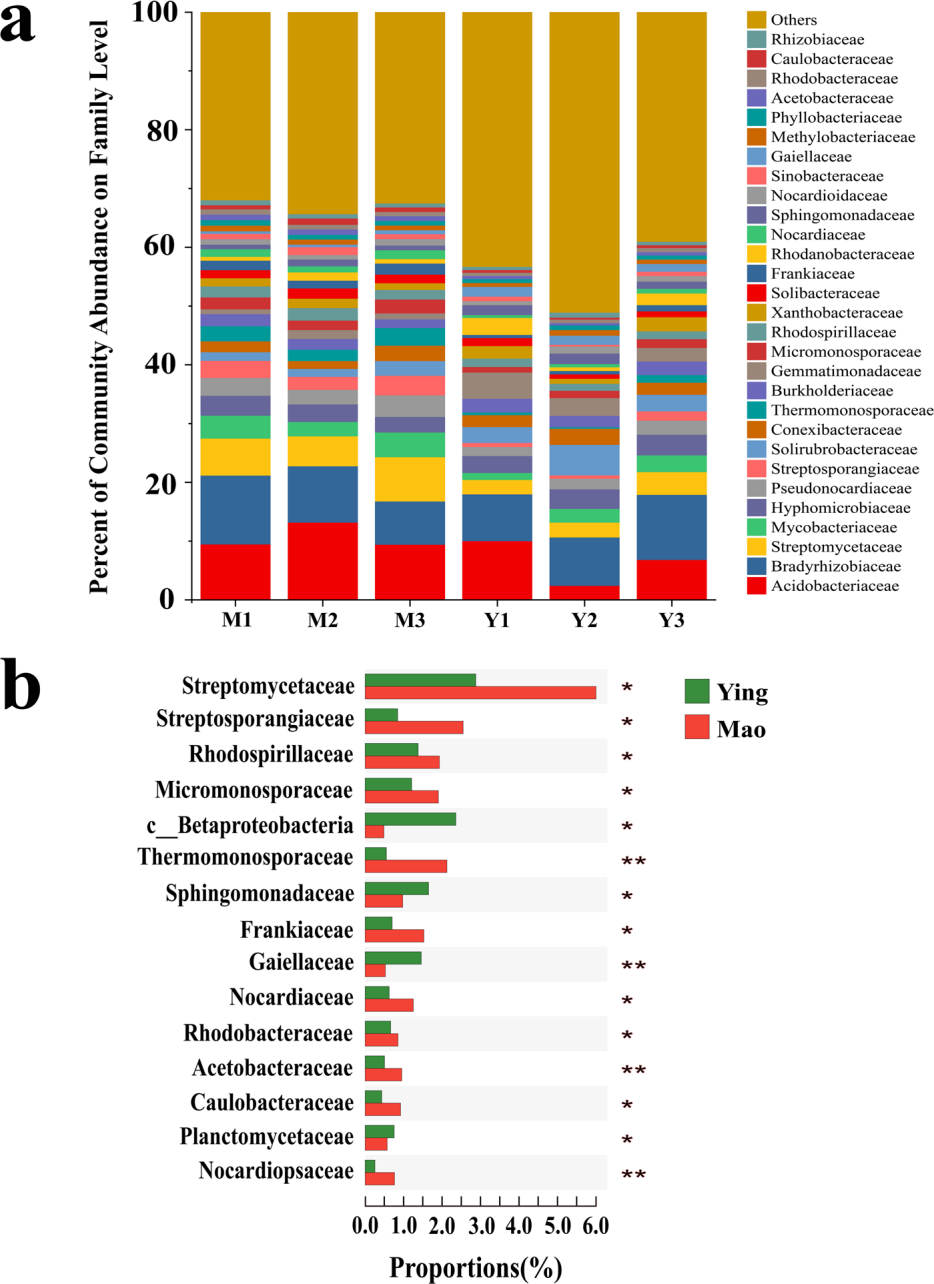
A comparison of procaryotic communities in the rhizosphere. (a) Relative abundance of the most abundant prokaryote at the family level. (b) Significant discrepancies in indicator prokaryotes between Mao and Ying groups at the family level. *P ≤ 0.05, **P ≤ 0.01.

The main families included *Acidobacteriaceae* (9.57%), *Bradyrhizobiaceae* (9.16%), *Streptomycetaceae* (5.41%), *Mycobacteriaceae* (3.10%), and *Hyphomicrobiaceae* (3.03%) (Figure 3a). The families *Streptomycetaceae*, *Streptosporangiaceae*, *Rhodospirillaceae*, *Micromonosporaceae*, and *Thermomonosporaceae* were significantly more abundant in the Mao sample than in the Ying sample (*P* ≤ 0.05). The abundances of the families *Sphingomonadaceae*, *Gaiellaceae*, and *Planctomycetaceae* were significantly higher in the Ying sample than in the Mao sample (Figure 3b).

**Figure 4:**
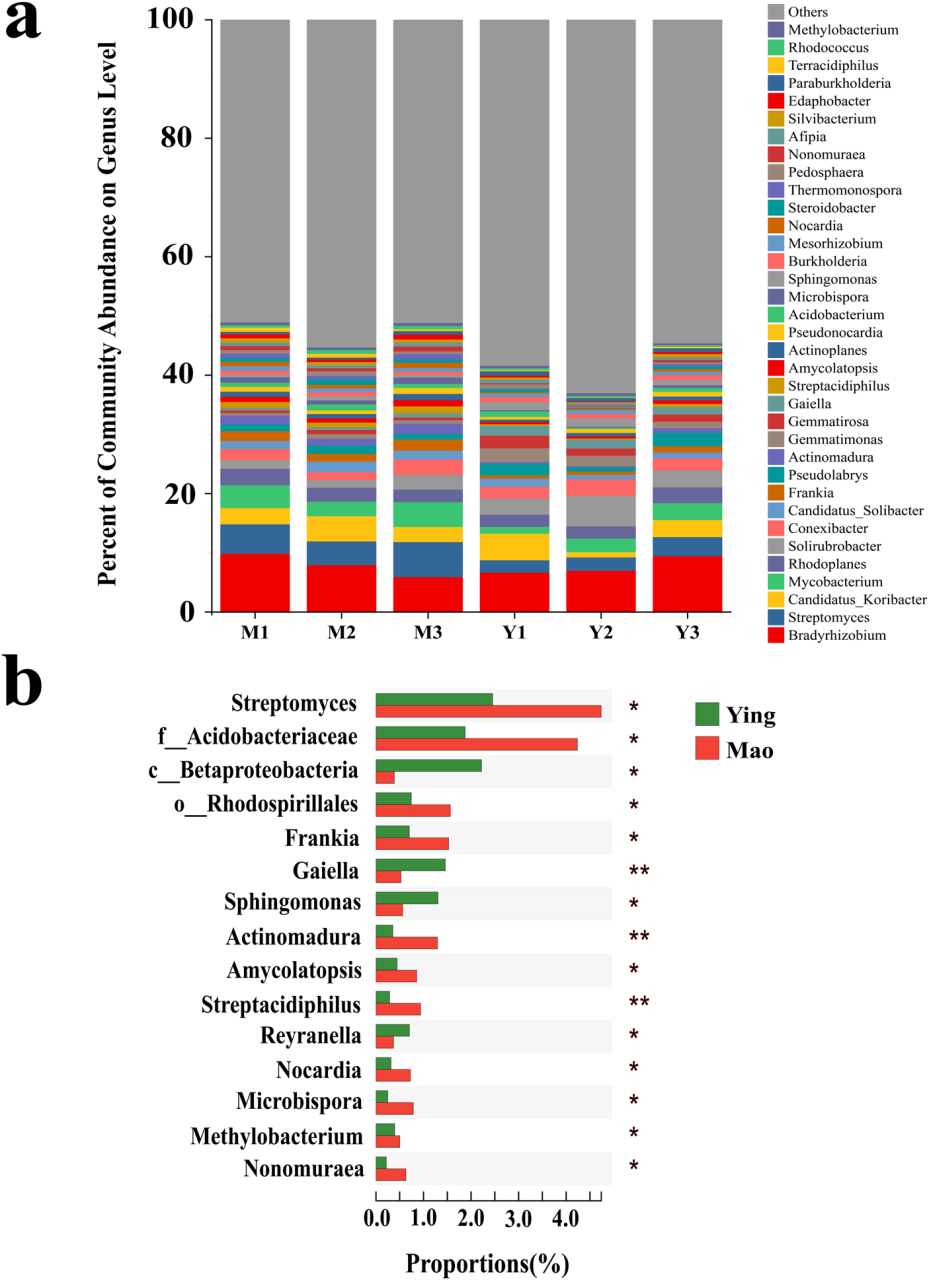
A comparison of procaryotic communities in the rhizosphere. (a) Relative abundance of the most abundant prokaryote at the genus level. (b) Significant discrepancies in indicator prokaryotes between Mao and Ying groups at the genus level. *P ≤ 0.05, **P ≤ 0.01.

At the genus level, *Bradyrhizobium* (7.62%), *Streptomyces* (4.30%), *Candidatus_Koribacter* (3.15%), *Mycobacterium* (3.10%), and *Rhodoplanes* (2.33%) were the top five abundant genera across all communities (Figure 4a). Genera with significant differences between the Mao and Ying samples included *Streptomyces*, *Frankia*, *Gaiella*, *Sphingomonas*, *Actinomadura*, and *Strepta cidiphilus* (Figure 4b). A total of 2,817 genera were identified in all samples, and those with abundances less than 0.01 were combined into the others.

### 3.4 Characteristic analysis of the core rhizosphere microbiome of *A. lancea*

**Figure 5:**
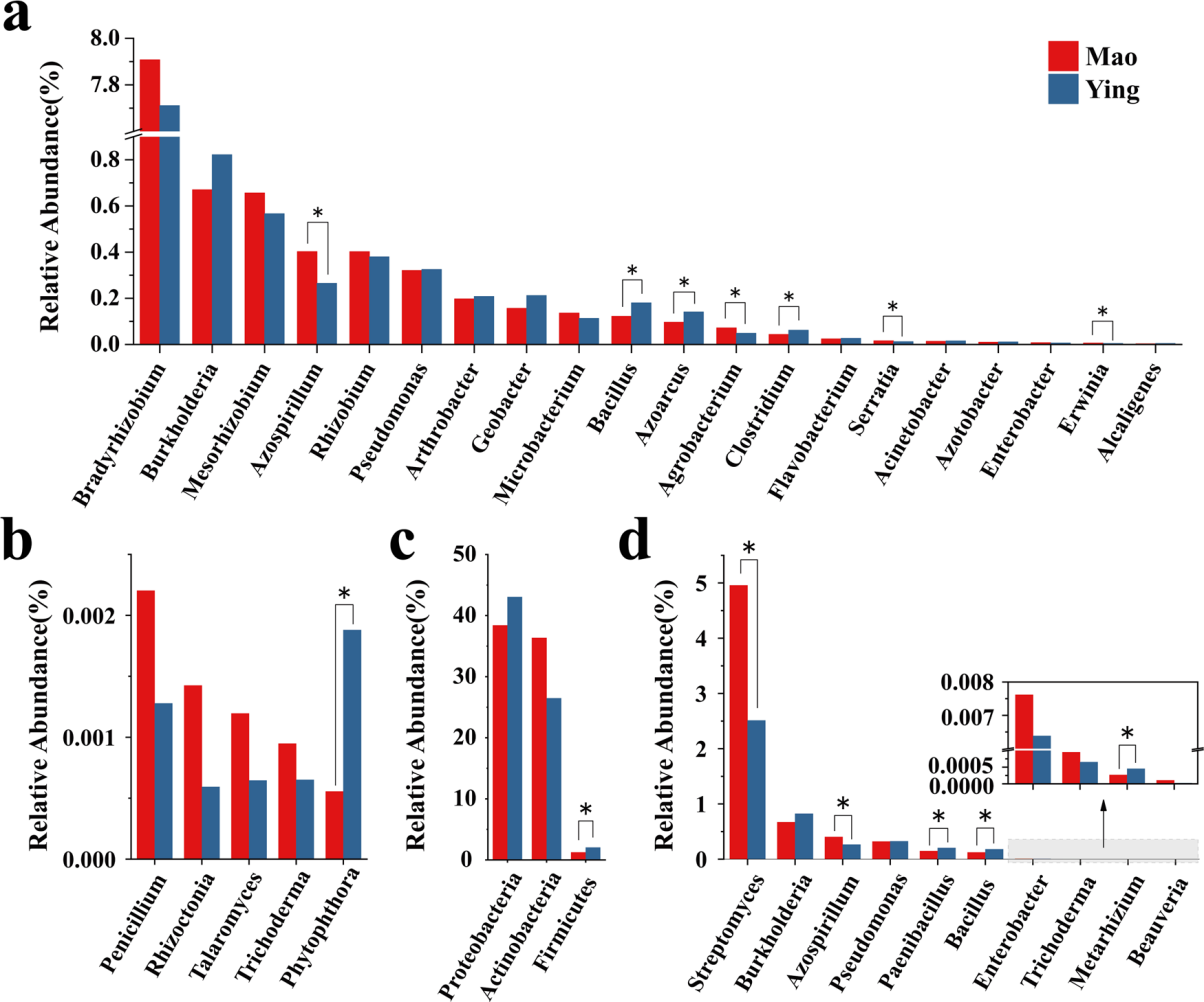
Detailed relative abundance of the core rhizosphere microbiome involved in (a) plant growth-promoting rhizobacteria, (b) plant growth-promoting fungi, (c) disease suppression, and (d) biological control agent. *P ≤ 0.05.

Plant growth-promoting rhizobacteria refer to microorganisms that reside in the plant’s rhizosphere and rhizoplane that can directly or indirectly improve the growth conditions and quality of the host plant. Their main functions are nitrogen fixation, phosphorus solubilization, secretion of plant growth-promoting substances, and synthesis of iron production carriers [26]. The results of the independent sample *T*-test showed that there were significant differences in the relative abundances of rhizosphere core microorganisms of *A. lancea* between the two sites. The abundance of *Bradyrhizobium* in the rhizosphere of *A. lancea* was the highest (more than 7%), and its abundance in the Mao sample was higher than that of the Ying sample, followed by *Burkholderia* and *Mesorhizobium*. Moreover, the abundances of *Azospirillum*, *Agrobacterium*, *Serratia*, and *Erwinia* were higher in the Mao sample than in the Ying sample (Figure 5a). *Azospirillum* and *Erwinia* belong to asybiotic nitrogen-fixing bacteria, which not only promote root growth but also transport more water and minerals to the above-ground parts of the host plant. These bacteria are often used in the production of food crops because they do not need to form nodules with plants to perform biological nitrogen fixation [27].

Plant growth-promoting fungi are microorganisms that reside in the rhizosphere soil of various plants. They perform the following functions in plants: biocontrol potential through competition for nutrients, growth hormone production, and mineral solubilization [28]. Plant growth-promoting fungi in the *A. lancea* rhizosphere include *Penicillium*, *Rhizoctonia*, *Talaromyces*, *Trichoderma*, and *Phytophthora*. *Penicillium* had the highest relative abundance in the Mao sample, followed by *Rhizoctonia*, *Talaromyces*, and *Trichoderma*. The abundance of *Penicillium* in the Mao sample was 1.7 times higher than that in the Ying sample (Figure 5b).

Multiple disease suppression prokaryotes were observed in the rhizosphere of *A. lancea*, such as Proteobacteria, Actinobacteria, and Firmicutes (Figure 5c). Furthermore, ten biological control genera were identified (Figure 5d) [29]. *Streptomyces*, the relative abundance of which was 1.9 times higher in the Mao sample than in the Ying sample, can produce a variety of antibiotics to inhibit the growth and reproduction of plant pathogens and perform biological control functions via hyperparasitism, the production of secondary metabolites, and induction of plant resistance [30].

### 3.5 Environmental factors and their correlations with the microbial community

**Figure 6:**
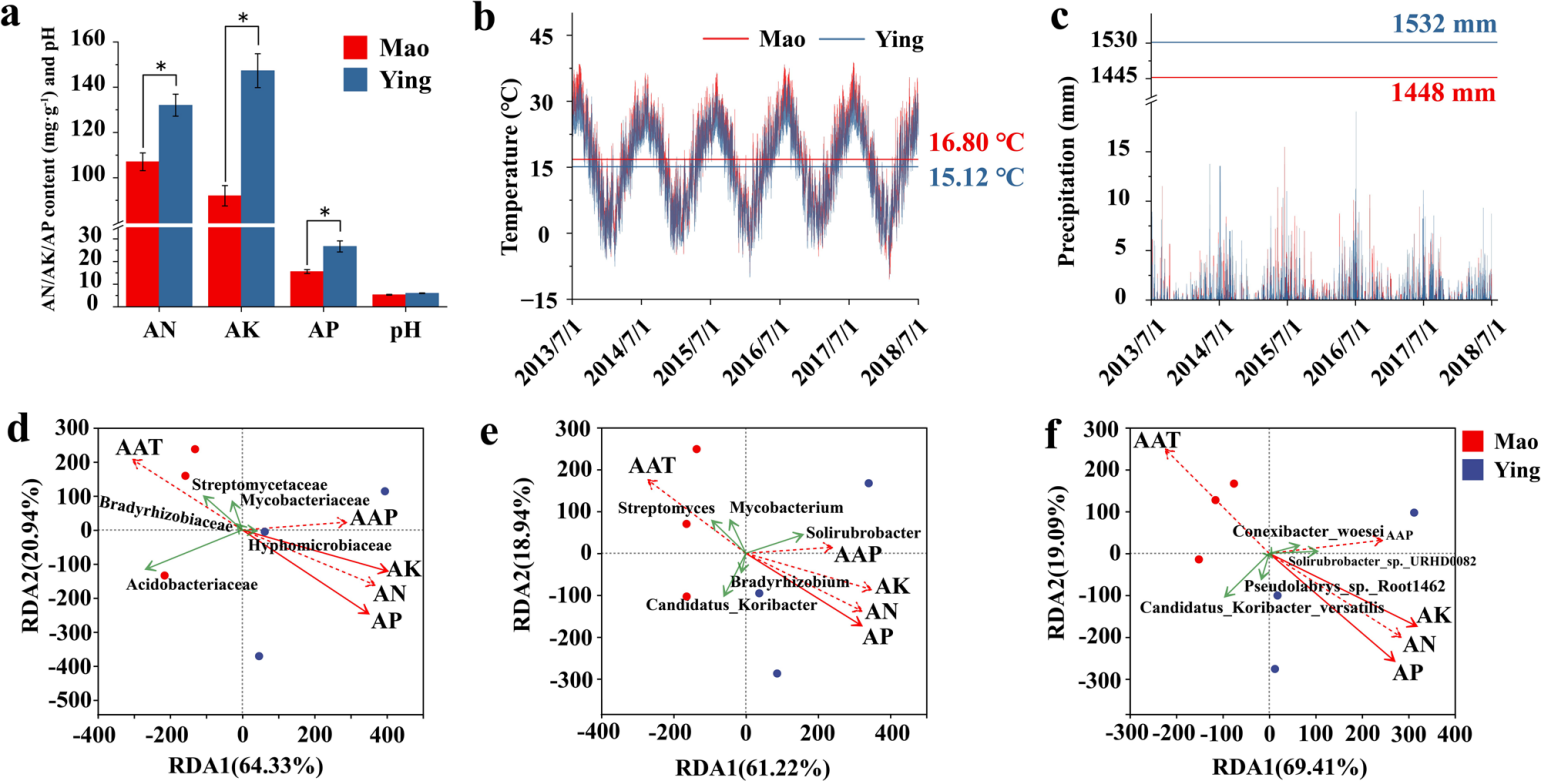
Differences in the environmental factors between groups, including (a) AN, AP, AK, and pH; (b) AAT; and (c) AAP. (d–f) RDA for rhizosphere microbes with environmental factors at the family, genus, and species taxonomic levels. The solid line represents environmental factors with significance to indicators, while the dotted line represents non-significance. *P ≤ 0.05.

Previous studies have shown that habitat and soil type, rather than host genetic background, have greater effects on the overall structure of the microbiome. However, the habitat and soil characteristics that have specific effects on the microbial community remain unclear. The test results for the soil physicochemical factors are shown in Figure 6a. There were significant differences in the AN, AP, and AK contents between the Mao and Ying samples. The AAT at the Mao site was significantly higher than that at the Ying site; however, there was no significant difference in the AAP between them (Figure 6b and c). According to the RDA based on the environmental factors and the most abundant prokaryotes among the samples, the two groups were separated obviously at the family, genus, and species levels (Figure 6d–f). At the family level, RDA1 and RDA2 explained 64.33 and 20.94% of the variance values, respectively. AAP and AK were the main influencing factors for RDA1. At the genus level, the abundance of *Streptomyces* and *Mycobacterium* correlated positively with the AAT, while *Solirubrobacter* correlated positively with the AAP. At the species level, RDA1 and RDA2 explained 64.91 and 19.09% of the variance values, respectively. AP and AK correlated significantly with the changes in rhizosphere microbial community (Table 1). In particular, AP had significant effects on the rhizosphere microbial community structure of *A. lancea* at the three taxonomic levels.

**Table 1:** The RDA results of environmental factors and microbes (values in bold mean significant, *P* ≤ 0.05)

Environmental factors	Family level		Genus level		Species level	
	*R* ^2^	*P*	*R* ^2^	*P*	*R* ^2^	*P*
AN	0.80446	0.08194	0.83882	0.07917	0.84386	0.09583
AP	0.91162	**0.01806**	0.90959	**0.0375**	0.97179	**0.01111**
AK	0.88133	**0.03194**	0.88041	0.0625	0.91693	**0.04722**
AAT	0.67033	0.18056	0.71339	0.14444	0.78061	0.10278
AAP	0.41188	0.42083	0.38662	0.4875	0.41664	0.45972

Phosphorus is one of the three essential elements for plant growth and is involved in the biosynthesis of many macromolecular substances such as nucleic acids and proteins. If the phosphorus supply is insufficient, plant growth will be inhibited, which will affect the absorption of other elements, leading to developmental delays, leaf shedding, and lower yields [31]. Plants mainly absorb water-soluble phosphorus from inorganic phosphorus in the soil, which leads to a low utilization efficiency of soil phosphorus [32]. Phosphate-solubilizing microorganisms (PSMs) are a group of microorganisms capable of hydrolyzing organic and inorganic phosphorus into soluble forms, thus increasing their bioavailability to plants. PSMs are quite abundant in the soil and are commonly associated with the rhizosphere of plants. There is also great diversity in PSMs, including *Azospirillum*, *Bacillus*, *Pseudomonas*, and *Nitrosomonas* [33], which can hydrolyze organophosphorus and inorganic phosphorus into soluble form by secreting extracellular enzymes or releasing phosphorus during substrate degradation, thus increasing the content of AP in the soil.

### 3.6 Function potential analysis of the microbiome

**Figure 7:**
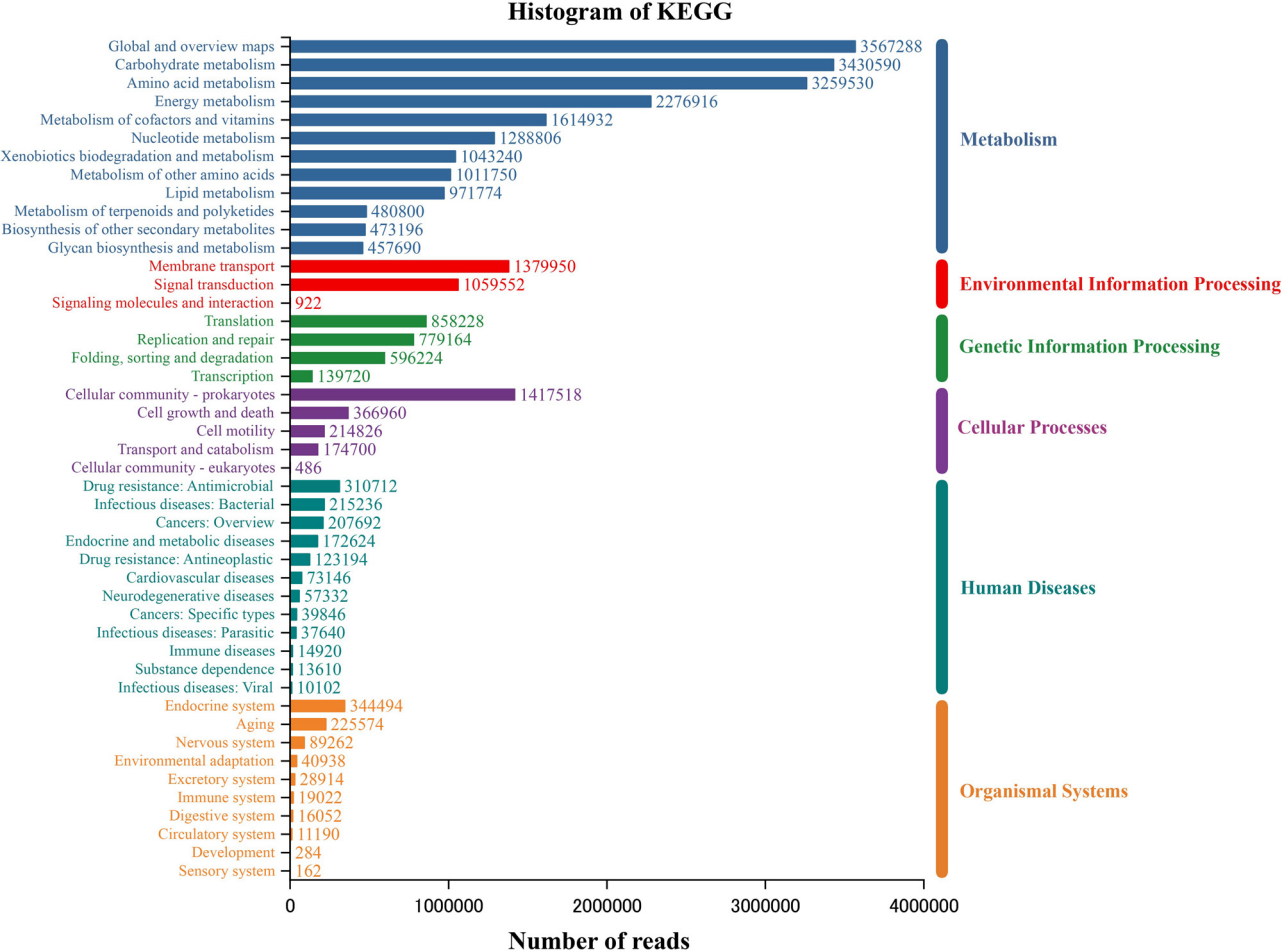
The annotated statistical map of the KEGG function of the rhizosphere microbiome of *A. lancea*.

The main COG functional annotations of the six rhizosphere microbiome samples were amino acid transport and metabolism (9.76%), energy production and conversion (8.00%), carbohydrate transport and metabolism (6.15%), signal transduction mechanisms (6.08%), and replication, recombination, and repair (6.03%). A total of 6,730 KEGG orthologies were obtained from all six samples, participating in 385 KEGG pathways (level 3) according to their KEGG functional annotations. Metabolism (72.23%) was the most abundant function pathway at level 1, followed by genetic information processing (7.85%), environmental information processing (6.77%), cellular processes (6.18%), human diseases (4.10%), and organismal systems (2.88%) (Figure 7). The genes enriched in the top 40 KEGG pathways (level 3) accounted for 61% of the total genes. Overall, these results provided an overview of the rhizosphere microbiome function of *A. lancea*.

**Figure 8:**
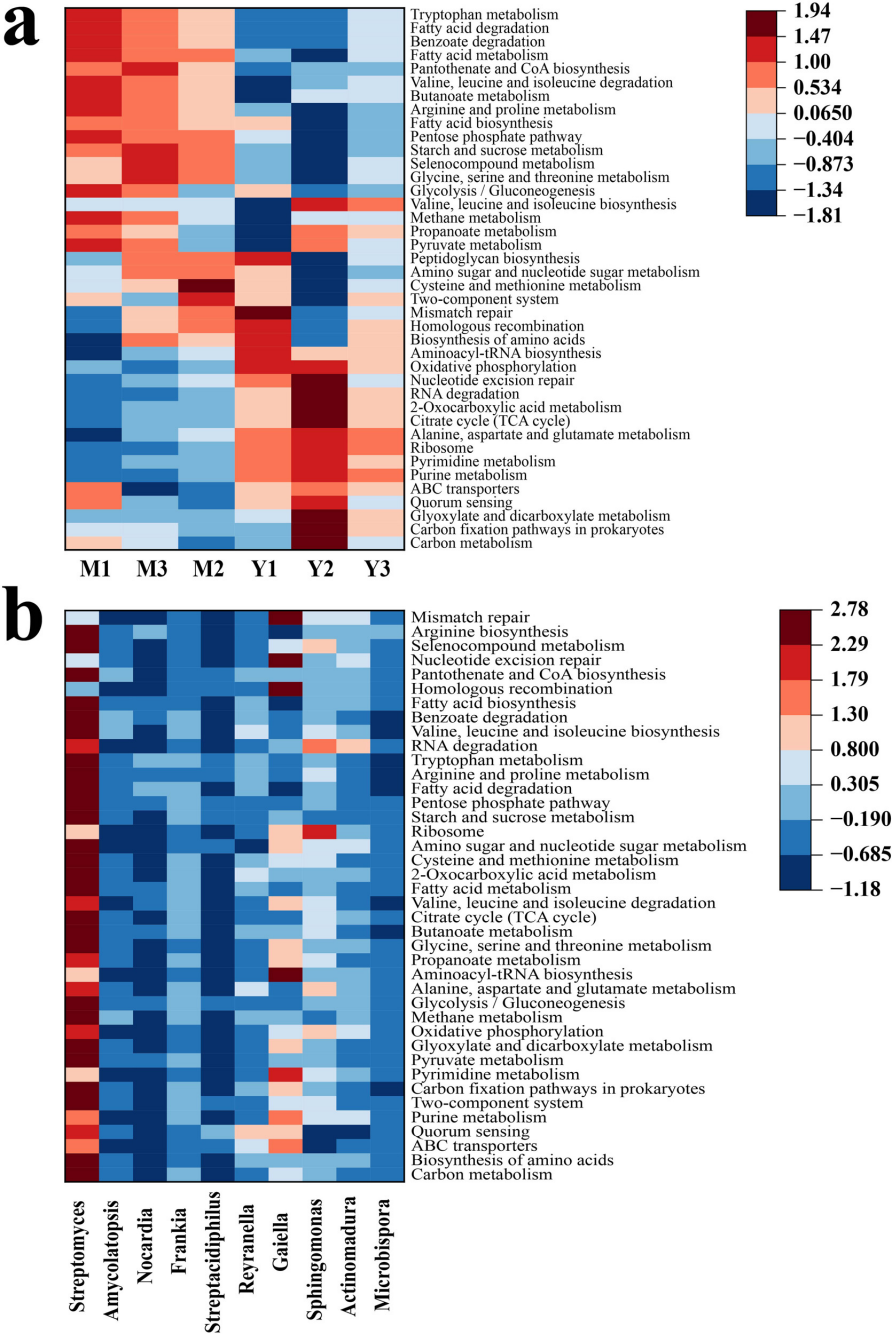
(a) Heat map analysis of the top 40 most abundant KEGG functional pathways using normalized abundance. The heat map is color-coded based on *z*-scores (zero mean normalization). (b) Pearson correlation analysis between genera with significant difference between Mao and Ying and metabolic pathway on level 3.

Plant–microbe and microbe–microbe interactions are likely to be important factors for the aggregation of the rhizosphere microbiome. The Mao and Ying samples were enriched and compared in different metabolic pathways (Figure 8a). The two-component system, a metabolic pathway known to be involved in plant–microbe and microbe–microbe interactions, had a higher proportion in the Mao sample than in the Ying sample. The two-component system regulates the majority of bacterial physiological functions, including chemotaxis, which makes bacteria more congregate. It may mean that the rhizosphere environment of *A. lancea* in Maoshan is more conducive to microbial aggregation. Tryptophan is not only an important amino acid for protein biosynthesis but also an important precursor for auxin biosynthesis. Tryptophan metabolism was enriched in the Mao sample compared with that in the Ying sample. We also found that compared with those in the Ying sample, the Mao sample had a greater proportion of amino acid anabolism pathways, including glycine, serine, threonine, valine, leucine, isoleucine, cysteine, methionine, arginine, and proline metabolism. The results indicated that the microbes in the Mao rhizosphere potentially could not obtain enough nutrients directly from root exudates of *A. lancea*, and needed to synthesize amino acids themselves or via other related ways. Subsequently, the 10 microbial genera with significant differences between the samples and the top 40 KEGG functional pathways were selected for functional contribution analysis (Figure 8b). The results showed that *Streptomyces* was the main driving factor of the two-component system and tryptophan metabolism, which was enriched in the Mao sample. In addition, the functional pathways affected by *Streptomyces* included carbon metabolism, biosynthesis of amino acids, carbon fixation pathways in prokaryotes, and pyruvate metabolism.

### 3.7 Correlation analysis of medicinally active ingredients with the rhizosphere microbiome

**Figure 9:**
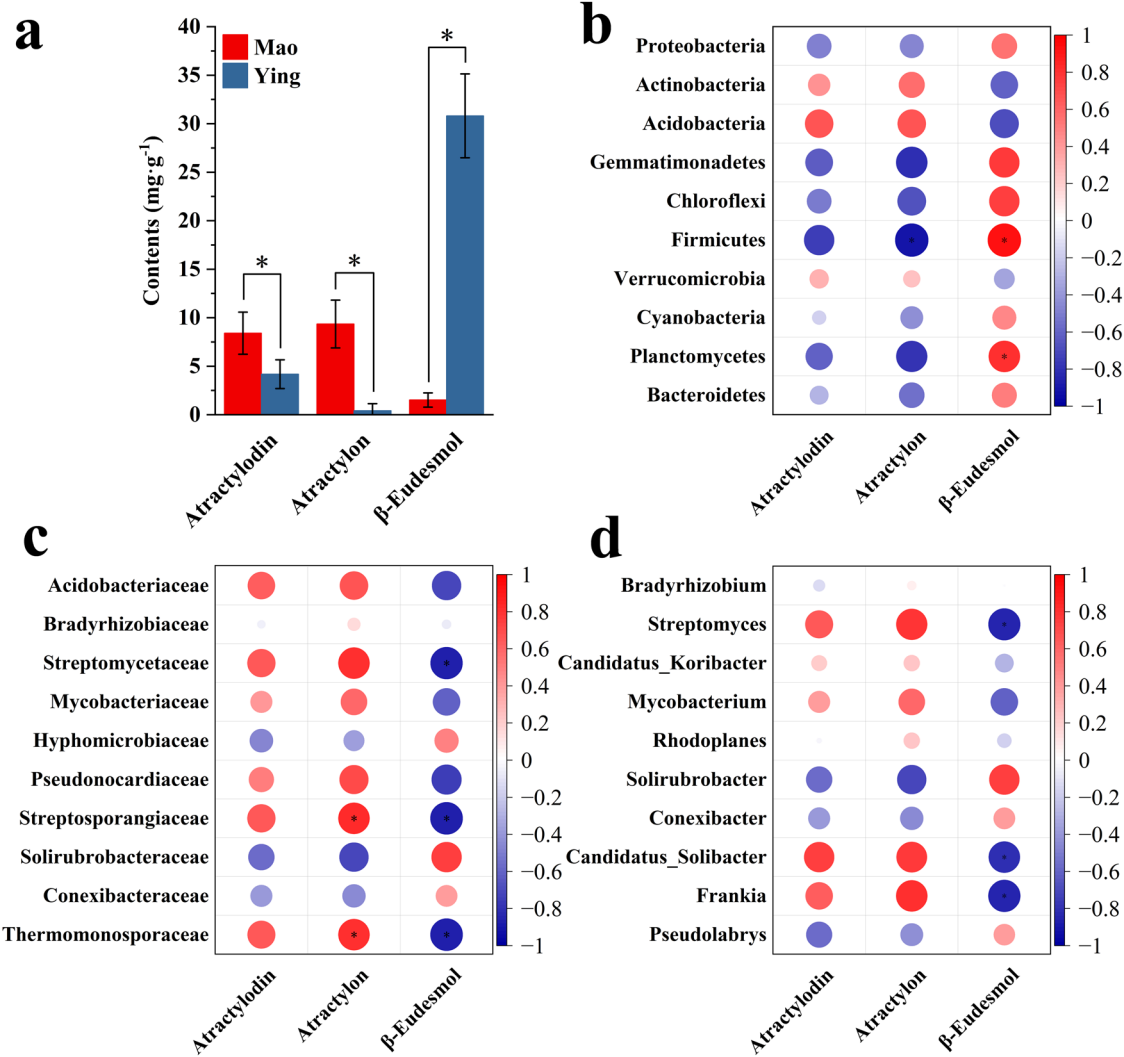
(a) Contents of atractylodin, atractylon, and β-eudesmol in the Mao and Ying samples. Correlation analysis between medicinally active ingredients of *A. lancea* and the rhizosphere microbiome at phylum (b), family (c), and genus (d) levels. *P ≤ 0.05.

Rhizosphere microbes improve the utilization rate of nutrients in the soil, thereby affecting the growth and metabolic processes of medicinal plants, ultimately affecting the efficacy of medicinal materials [34]. The contents of atractylodin, atractylon, and β-eudesmol in *A. lancea* from the two regions were determined (Figure 9a). The results showed that the contents of atractylodin and atractylon in the Mao sample were significantly higher than those in the Ying sample, while the content of β-eudesmol was higher in the Ying sample. The results showed that there were differences in the active ingredients between the genuine producing area and the non-genuine producing area, which was consistent with the results of previous studies [35]. We performed a correlation analysis between the three medicinally active ingredients and the top ten abundant microbes at the phylum, family, and genus levels. At the phylum level (Figure 9b), the content of atractylodin and atractylon correlated positively with the abundance of Actinobacteria, Acidobacteria, and Verrucomicrobia, while the content of β-eudesmol correlated significantly and positively with the abundance of Firmicutes and Planctomycetes. At the family level (Figure 9c), the atractylon content correlated significantly with the abundance of *Streptosporangiaceae* and *Thermomonosporaceae*. At the genus level (Figure 9d), the content of β-eudesmol correlated positively with the abundance of *Solirubrobacter*, *Conexibacter*, and *Pseudolabrys*. In summary, microbes might play a very important role in plant secondary metabolism synthesis.

## 4 Discussion

### 4.1 Differences in the microflora composition between different regions of *A. lancea*

Through high-throughput sequencing, researchers found that the rhizosphere microorganisms of *A. lancea* were abundant and the relationship between microorganisms and plants was complex [21]. Furthermore, to the best of our knowledge, this is the first metagenomic report on rhizosphere microorganisms of *A. lancea* in different regions, and the quality is high compared with other studies. Similar to studies of medicinal plants such as *A. membranaceus* and *Alisma*, we also found that the composition and diversity of rhizosphere microbial communities are influenced by various environmental factors, including AN, AP, AK, and pH [36,37]. Although the contents of nutrients (AN, AP, and AK) in the Yingshan soil were significantly higher than those of the Maoshan soil, the α-diversity results showed no significant difference in the richness and diversity of the rhizosphere microbial communities between these two areas. PCoA could distinguish the two groups of samples, which means that soil and climate factors in this study had a greater impact on the dominant microbial flora than community diversity. Actinobacteria and Proteobacteria were the dominant phyla in the Mao and Ying samples, respectively. Studies have shown that the same species of plants recruit similar microorganisms to form rhizosphere environments on different soils [38]. From the perspective of genera, it can be seen that the abundance of most rhizosphere microorganisms in *A. lancea* is less than 0.01 (Figure 4a) but a rich and stable rhizosphere microbiome is formed.

In addition, *Glomus*, a group of arbuscular mycorrhizal fungi, was annotated in the Mao sample but not in the Ying sample. Studies have shown that inoculation of arbuscular mycorrhizal fungi could improve biomass, promote the accumulation of essential oil components, and reduce the occurrence of root rot of *A. lancea* [39,40], which might be one of the reasons for the better quality of medicinal materials in the Mao sample than in the Ying sample.

### 4.2 *A. lancea* contain various core rhizosphere microbiomes

The abundance of the prokaryotic community in each sample was above 99%; therefore, it was the dominant community in the rhizosphere soil of *A. lancea*. The samples contained 0.6% of archaea, which is an important component of the plant microbiome [41,42]. The main feature that distinguishes archaea from other microorganisms is that they can adapt to survive in extreme environments; therefore, they might help plants by enhancing their immune responses and adaptation to abiotic stresses [43]. Moreover, archaea are also involved in environmental nutrient cycling in plant ecosystems [44], and some archaea are also considered as plant growth-promoting archaea [45]. The identification of a certain abundance of archaea among the rhizosphere microbes of *A. lancea* is important for future studies on plant–archaeal interactions.

*Bradyrhizobium* (a type of plant growth-promoting rhizobacteria), which was more abundant in the Mao sample than in the Ying sample, can fix nitrogen in the environment and supply it to plants, thereby promoting plant growth directly or indirectly [46]. *A. lancea* plantations are faced with multiple challenges, including the impedance of growth by heat stress and drought [47,48]. *Actinomadura* and *Acrophialophora*, which are more abundant in the Mao samples, have been shown to increase *A. lancea* systemic tolerance to abiotic stresses, such as drought, thereby affecting the growth and accumulation of medicinal compounds [49]. In the rhizosphere of *A. lancea*, *Penicillium* in the Mao sample had the highest abundance. The biomass of *A. lancea* is affected by *Streptomyces* and other microorganisms [50]. *Streptomyces* is abundant in soil and contains many secondary metabolic biosynthesized gene clusters, such as that for abamectin synthesis, which has a strong effect on controlling plant diseases and insect pests [51,52]. We found that *Streptomyces* made the highest contribution to the functional difference of the rhizosphere microbiome between the two areas, and the abundance of *Streptomyces* in the Mao sample was significantly higher than that in the Ying sample.

### 4.3 Relationship between rhizosphere microorganisms and medicinally active ingredients

The main active ingredients of *A. lancea* are atractylodin, atractylon, and β-eudesmol. Atractylodin is a polyalkyne compound, while atractylon and β-eudesmol are sesquiterpenoids. Atractylodin is the quality control index of atractylodis rhizoma (Chinese herbal medicine) in Chinese Pharmacopoeia (2020). The significant difference in the content of the three components between the Mao and Ying samples might also be closely related to the rhizosphere microorganisms. The enrichment of metabolites secreted by rhizosphere microorganisms through plant roots is common in the growth and development of medicinal plants. Conversely, rhizosphere microorganisms can also increase the synthesis and accumulation of secondary metabolites by regulating nutrients and the activities of key enzymes to synthesize active ingredients [53,54]. We found that the contents of atractylodin and atractylon correlated positively with the abundance of *Streptomyces*, *Candidatus-Solibacter*, and *Frankia*. The content of β-eudesmol correlated positively with the abundance of *Solirubrobacter* in soil, suggesting that these microorganisms were directly or indirectly involved in the synthesis of secondary metabolites. The *Streptomyces* genus has been a rich source of bioactive natural products, medicinal chemicals, and novel drugs [55]. Sesquiterpenoids synthesized by *Streptomyces* species include roseosporol A and albaflavenoid [56,57].

## 5 Conclusions

There were differences in the diversity, structure, and function of the rhizosphere microbial communities of *A. lancea* in the genuine producing area and the non-genuine producing area. There are various reasons for these differences and not only soil factors. The differences in rhizosphere microorganisms might lead to a difference in the quality of *A. lancea*. The rhizosphere microorganisms of *A. lancea* are rich in species, some of which have great potential as the key microorganisms for disease prevention and control, stress response, and quality improvement, thus providing a valuable foundation for high-quality planting of *A. lancea* in the future.

## Supplementary Material

Supplementary Figure
