# Supplementary material for: Comparative analysis of the rhizosphere microbiome and medicinally active ingredients of Atractylodes lancea from different geographical origins
Source: Open Life Sci. 2023 Nov 23;18(1):20220769. doi: 10.1515/biol-2022-0769 (PMC10668115; doi:10.1515/biol-2022-0769)
Supplement: Supplementary Figure [file biol-2022-0769-sm.pdf]

Supplementary material

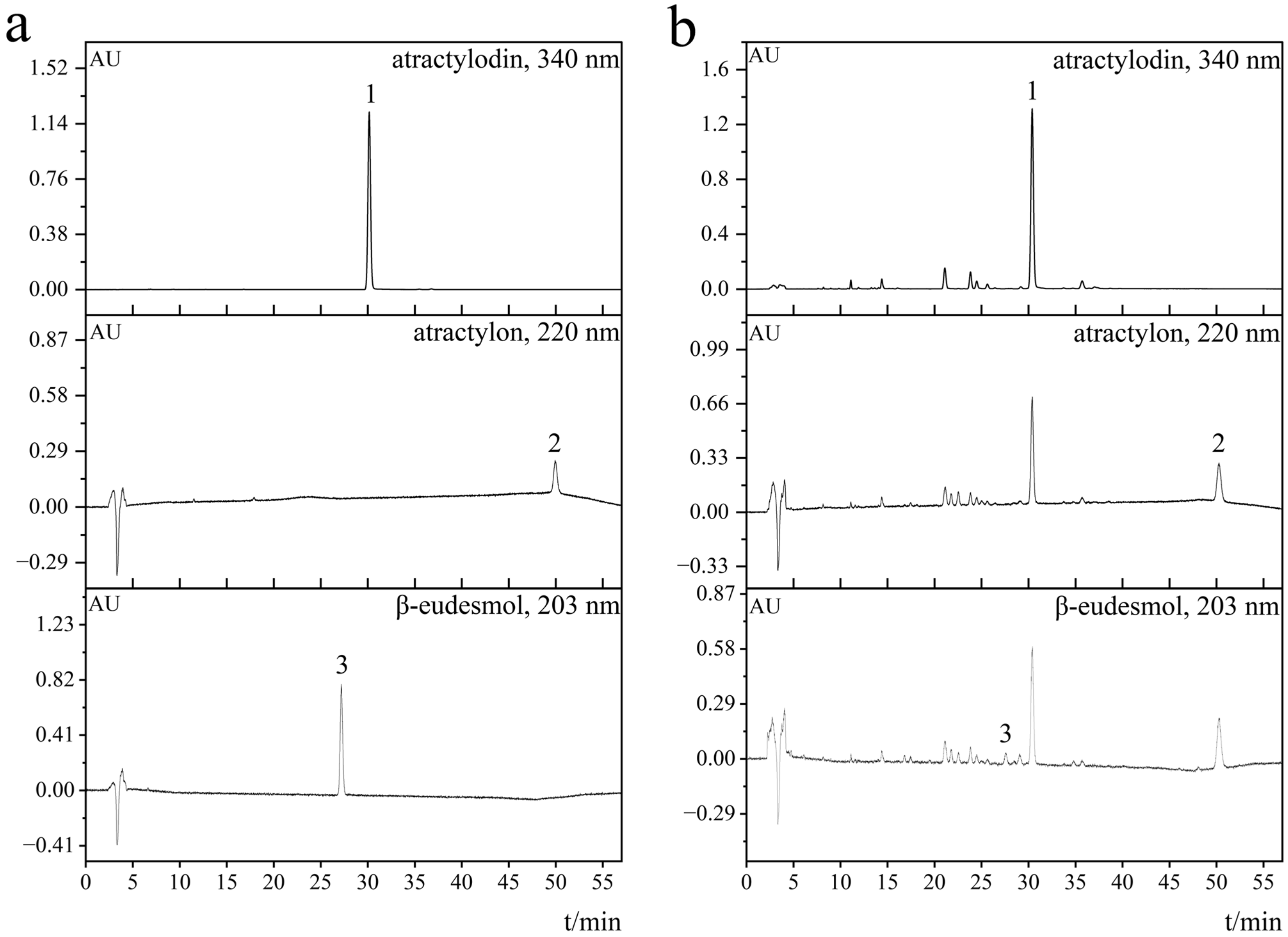

Figure S1: The HPLC chromatogranms of three active ingredients in *A. lancea*. (a) The chromatograms of standards of three active ingredients. (b) The representative chromatograms of three active ingredients of *A. lancea* from the Mao sample. 1: atractylodin, 2: atractylon, 3: β-eudesmol.

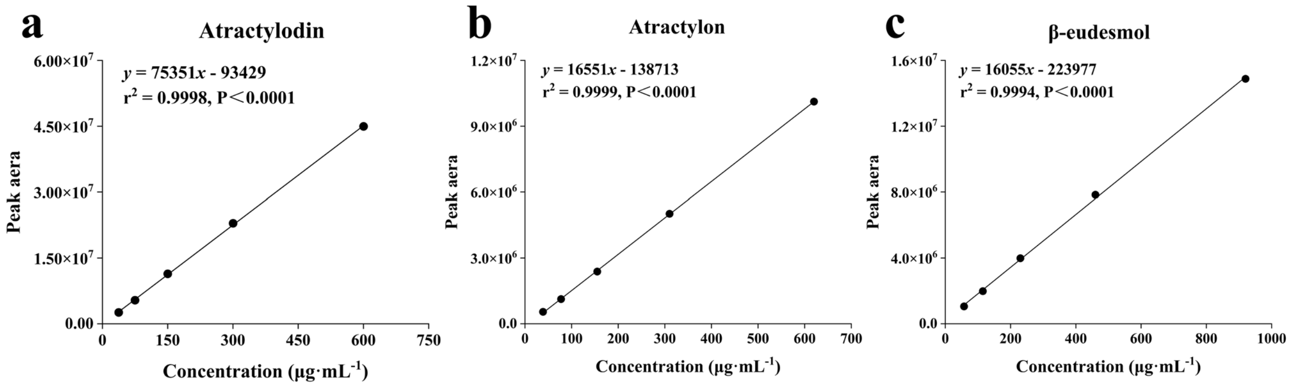

Figure S2: The HPLC standard curves of three active ingredients in roots of *A. lancea* were calculated using linear regression. (a) atractylodin, (b) atractylon, (c) β-eudesmol.
